# STAT3 exacerbates survival of cancer stem-like tumorspheres in EGFR-positive colorectal cancers: RNAseq analysis and therapeutic screening

**DOI:** 10.1186/s12929-018-0456-y

**Published:** 2018-08-02

**Authors:** Chun-Chia Cheng, Po-Nien Liao, Ai-Sheng Ho, Ken-Hong Lim, Jungshan Chang, Ying-Wen Su, Caleb Gon-Shen Chen, Ya-Wen Chiang, Bi-Ling Yang, Huan-Chau Lin, Yu-Cheng Chang, Chun-Chao Chang, Yi-Fang Chang

**Affiliations:** 1 Division of Hematology and Oncology, Department of Internal Medicine, Mackay Memorial Hospital, Taipei, Taiwan; 2 Laboratory of Good Clinical Research Center, Department of Medical Research, Mackay Memorial Hospital, Tamsui District, New Taipei City, Taiwan; 3 Division of Gastroenterology, Cheng Hsin General Hospital, Taipei, Taiwan; 4 Graduate Institute of Medical Sciences, School of Medicine, College of Medicine, Taipei Medical University, Taipei, Taiwan; 5 Division of Gastroenterology and Hepatology, Department of Internal Medicine, Taipei Medical University Hospital, Taipei, Taiwan; 6 Division of Gastroenterology and Hepatology, Department of Internal Medicine, School of Medicine, College of Medicine, Taipei Medical University, Taipei, Taiwan; 7 Department of Medicine, MacKay Medical College, New Taipei City, Taiwan

**Keywords:** Cancer stem cell, EGFR, LGR5, PDGFA, STAT3, Wnt

## Abstract

**Background:**

Cancer stem cells are capable of undergoing cell division after surviving cancer therapies, leading to tumor progression and recurrence. Inhibitory agents against cancer stem cells may be therapeutically used for efficiently eradicating tumors. Therefore, the aim of this study was to identify the relevant driver genes that maintain cancer stemness in epidermal growth factor receptor (EGFR)-positive colorectal cancer (CRC) cells and to discover effective therapeutic agents against these genes.

**Methods:**

In this study, EGFR-positive cancer stem-like cells (CSLCs) derived from HCT116 and HT29 cells were used as study models for in vitro inductions. To identify the differential genes that maintain CSLCs, RNAseq analysis was conducted followed by bioinformatics analysis. Moreover, a panel containing 172 therapeutic agents targeting the various pathways of stem cells was used to identify effective therapeutics against CSLCs.

**Results:**

RNAseq analysis revealed that 654 and 840 genes were significantly upregulated and downregulated, respectively, in the HCT116 CSLCs. Among these genes, notably, *platelet-derived growth factor A (PDGFA)* and *signal transducer and* *activator of transcription 3 (STAT3)* were relevant according to the cancer pathway analyzed using NetworkAnalyst. Furthermore, therapeutic screening revealed that the agents targeting STAT3 and Wnt signaling pathways were efficient in reducing the cell viabilities of both HCT116 and HT29 cells. Consequently, we discovered that STAT3 inhibition using homoharringtonine and STAT3 knockdown significantly reduced the formation and survival of HT29-derived tumorspheres. We also observed that STAT3 phosphorylation was regulated by epidermal growth factor (EGF) to induce PDGFA and Wnt signaling cascades.

**Conclusions:**

We identified the potential genes involved in tumorsphere formation and survival in selective EGFR-positive CRCs. The results reveal that the EGF-STAT3 signaling pathway promotes and maintains CRC stemness. In addition, a crosstalk between STAT3 and Wnt activates the Wnt/β-catenin signaling pathway, which is also responsible for cancer stemness. Thus, STAT3 is a putative therapeutic target for CRC treatment.

**Electronic supplementary material:**

The online version of this article (10.1186/s12929-018-0456-y) contains supplementary material, which is available to authorized users.

## Background

Colorectal cancer (CRC) is a leading cause of cancer-related morbidity and mortality worldwide. Early diagnosis followed by surgery can cure patients with CRC. Adjuvant chemotherapy is used for eradicating remnant tumor cells in high-risk CRC (stages II and III). Adjuvant therapy consists of either a single agent, such as capecitabine or 5FU/leucovorin, or a combination of agents, such as FOLFOX (leucovorin, 5FU, and oxaliplatin) or CapeOX (capecitabine and oxaliplatin). Moreover, chemotherapy with oxaliplatin, irinotecan, and 5FU/leucovorin and targeted therapy using epidermal growth factor receptor (EGFR) antibodies, such as cetuximab and panitumumab, are recommended for metastatic CRC [1–3]. However, many patients encounter cancer recurrence despite treatment. Because CRC recurrence is a major issue leading to poor survival rates, additional developments of novel therapeutics are necessary.

The literature indicates that cancer stem cells (CSCs) with higher self-renewal and pluripotency are responsible for tumor recurrence [4, 5] and that they are associated with metastatic CRC [6]. To the best of our knowledge, CSCs present high drug resistance [7, 8] and antiapoptosis property [9, 10] to survive tumor chemotherapies. Furthermore, CSCs induce the activation of oncogenic pathways, such as MET, HER2, and Wnt, for escaping the targeted therapies [11–15]. Therefore, to develop an effective therapy targeting specific driver genes, investigating the molecular mechanism of CSCs is essential [16]. Leucine-rich repeat-containing G-protein-coupled receptor 5 (LGR5) is a specific marker of CRC stem cells [16, 17], which participates in and activates the Wnt signaling pathway [18–20]. Activation of the Wnt signaling pathway is known for maintaining the survival of CRC stem cells. LGR5 is crucial during embryogenesis as a marker of adult intestinal stem cells in the small intestines [21]. Crypt base columnar (CBC) cells with high expression of LGR5 located interspersedly among the differentiated Paneth cells are capable of dividing into functional cells in the intestinal tissues [22]. The colorectal stem cells are similar to CBC cells that express high LGR5 [23]. Thus, Wnt signaling activation maintains the survival of CRC cells.

Because CRCs overexpress EGFR in > 90% of clinical patients, the literature indicates that EGFR is involved in activating the JAK–STAT signaling pathway [24] for the survival of CSCs [25, 26]. In fact, EGFR was reported to be responsible for maintaining the survival of cancer stem-like cells (CSLCs) in EGFR-positive cancers [27]. Therefore, EGFR downstream proteins, such as signal transducer and activator of transcription 3 (STAT3), play a crucial role in activating Wnt signaling in colon [28, 29] and ovarian [30] cancers. The crosstalk between STAT3 and the Wnt signaling pathway possibly occurs during miRNA-92 regulation [30]. STAT3 knockdown consequently reduces β-catenin expression in CRCs [29]. Furthermore, Wnt upregulates STAT3 for inhibiting cell differentiation in embryonic stem cells [31]. Additionally, suppression of STAT3 by its specific inhibitors [32, 33] leads to the prevention of Wnt signaling activation. The results demonstrate a crosstalk between STAT3 and the Wnt signaling pathway. In particular, β-catenin stabilized and activated by Wnt is predominantly detected in the invasive regions of colorectal carcinomas [34] where CSCs are responsible for tumor metastasis [35]. These pieces of evidence support that CSCs contribute to resistance against chemoradiotherapies, leading to tumor relapse [36]. Therefore, therapeutics that eradicate CSCs through targeting of specific driver genes are considered successful [6].

The aim of this study was to understand the potential molecular mechanisms of CSCs through RNAseq analysis followed by bioinformatics analysis for identifying the specific driver genes involved in the survival of cancer stem-like tumorspheres. This study also used in vitro therapeutic screening for identifying the targeted agents against EGFR-positive CRCs and their derived cancer stem-like tumorspheres. The CSLCs were derived from the colorectal HCT116 and HT29 cells through the addition of EGF, bFGF, insulin, and heparin in a serum-free cultured medium [37, 38]. Therefore, the genes involved in the EGF-, FGF-, and insulin-mediated signal transduction pathways were priorly investigated through RNAseq analysis. Furthermore, we conducted the study to identify the inhibitory agents against the driver genes in the CRC-derived tumorspheres and compare them with the results obtained from RNAseq and bioinformatics analyses.

## Methods

### Cell culture and tumorsphere formation

The colorectal HCT116 and HT29 cancer cells were gifted to us by the Institute of Nuclear Energy Research, Taiwan. They were free of *mycoplasma*. The HCT116 cells were cultured in McCoy’s 5A medium, and the HT29 cells were cultured in Dulbecco’s Modified Eagle’s Medium with 10% fetal bovine serum and 1% penicillin–streptomycin. Tumorsphere formation and measurement were the same as described previously [37]. Platelet-derived growth factor (PDGF)-AA was purchased from R&D Systems, Inc. (Minneapolis, MN, USA).

### EGFR measurement through flow cytometry

The obtained H520, HCT116, and HT29 cells were treated with 2 μg/mL of cetuximab–FITC for 2 h at 4 °C. Cetuximab–FITC was created and validated as in a previous study [39]. Subsequently, the medium was removed and the cells were washed thrice with phosphate-buffered saline (PBS). The cells in PBS buffer were analyzed for EGFR expression using a FACSCalibur Flow Cytometer (BD Bioscience, San Jose, CA, USA). In this study, H520 was used as an EGFR-negative cell line.

### Quantitative polymerase chain reaction

The procedure for mRNA extraction and complementary DNA preparation was the same as described previously [37]. Quantitative polymerase chain reaction (qPCR) was performed using the SYBR Green system (Applied Biosystems, Foster City, CA, USA) according to the manufacturer’s instruction. The primers are presented in Table 1.

**Table 1:** Primers used in this study

Gene	Direction	Sequence (5′ to 3′)
*LGR5*	Forward	CTCTTCCTCAAACCGTCTGC
	Reverse	GATCGGAGGCTAAGCAACTG
*CD133*	Forward	CTATTCAGGATATACTCTCAGCATT
	Reverse	TTTCTGTGGATGTAACTTTCAGTG
*PDGFA*	Forward	ACGTCAGGAAGAAGCCAAAA
	Reverse	GGCTCATCCTCACCTCACAT
*GAPDH*	Forward	GAGTCAACGGATTTGGTCGT
	Reverse	TTGATTTTGGAGGGATCTCG

### RNAseq and bioinformatics analyses

RNAseq analysis was performed to compare the differential levels of genes between the HCT116 cells and HCT116-derived tumorspheres using HiSeq 4000 with paired-end 150 bp sequencing. The differential genes are shown in Additional file 1: Table S1. The genes that showed a > 1-fold change (log2) in expression in the HCT116-derived tumorspheres compared with the parental HCT116 cells were classified according to their molecular function using the PANTHER classification system (http://pantherdb.org/). The genes that showed > 3-fold change (log2) in expression in the HCT116-derived tumorspheres are presented in Table 2. In addition, the genes classified using PANTHER that are associated with the EGF, FGF, and insulin pathways are presented in Table 3. Moreover, using the Kaplan–Meier plotter derived from the database PROGgeneV2 (http://watson.compbio.iupui.edu/chirayu/proggene/database/index.php), we identified the relationship between the overall survival rate and mRNA expression of the target genes. The protein–-protein interaction was analyzed using NetworkAnalyst (http://www.networkanalyst.ca/), and pathway activations were selected and matched according to the KEGG database.

**Table 2:** Genes that showed > 3-fold change (log2) in expression in HCT116-derived tumorspheres

Gene	fold chang (log2)	qvalue
*GBP2*	5.234	7.48E-05
*KRTAP2–3*	4.9065	1.31E-36
*LUCAT1*	4.8558	2.11E-10
*ITGAX*	4.66	4.87E-09
*TMEM158*	4.4984	3.71E-85
*CREB5*	4.4312	5.02E-10
*EFEMP2*	4.3996	1.17E-11
*ANKRD37*	4.3836	6.70E-13
*ANGPTL4*	4.3529	3.55E-103
*NDRG1*	4.1854	0
*IZUMO4*	4.0022	1.24E-12
*PMEPA1*	3.951	0.000462
*LINC00202–1*	3.9266	0.004557
*HLA-V*	3.9176	0.001176
*CXCL8*	3.8982	2.38E-20
*KB-1460A1.5*	3.7413	0.000276
*C1orf228*	3.657	0.002742
*IGFBP3*	3.5875	3.18E-09
*HIST1H1C*	3.5841	1.94E-18
*PDGFA*	3.5807	1.44E-48
*CITED4*	3.5739	0.000183
*CXCR4*	3.5015	0.003701
*STRC*	3.4502	0.004858
*ISG20*	3.3791	0.000322
*HILPDA*	3.3395	5.75E-36
*FAM83A*	3.339	0.00102
*AMY2B*	3.2986	0.001327
*ALDOC*	3.2823	1.74E-66
*AMN*	3.2771	0.000458
*YPEL3*	3.2485	0.00012
*SLPI*	3.2287	0.000305
*RP11-102 K13.5*	3.1821	0.000914
*CYP4F12*	3.177	1.68E-05
*AGPAT4*	3.163	5.97E-24
*CORIN*	3.111	0.00075

**Table 3:** Upregulated genes associated with EGF, FGF, and insulin pathways in HCT116-derived tumorspheres (analyzed using PANTHER, http://pantherdb.org/)

Pathway	Gene	fold chang (log2)	qvalue
EGF	*RRAS*	1.9498	6.33E-25
	*RAC2*	1.1094	4.70E-07
	*PIK3CD*	1.2025	0.002031
	*STAT2*	1.1837	7.66E-06
	*MAPK15*	1.2498	5.45E-07
	*PRKCG*	2.2982	0.0042873
	*SHC2*	2.6131	0.0019177
	*STAT3*	1.0825	1.18E-11
	*PPP2R5B*	2.4064	1.95E-14
	*SFN*	1.2086	2.92E-57
	*AREG*	1.6495	2.22E-52
FGF	*FGFR1*	1.0804	3.25E-10
	*RAC2*	1.1094	4.70E-07
	*PIK3CD*	1.2025	0.002031
	*PRKCG*	2.2982	0.0042873
	*PPP2R5B*	2.4064	1.95E-14
	*SFN*	1.2086	2.92E-57
Insulin	*IRS2*	2.0046	1.06E-07
	*FOXO3*	1.3511	9.38E-12
	*PDK1*	1.4964	0.00013109
	*PIK3CD*	1.2025	0.002031

### Cell viability

The alarmarBlue assay was used according to the manufacturer’s protocol to determine cell viability. Inhibitors against the HCT116 and HT29 cells were added to the cell culture and incubated for 48 h. Cell viability was then measured using alarmarBlue. STAT3 inhibitors against HT29CSCs were added to the culture and incubated for 7 days because tumorsphere formation requires > 7 days.

### Western blots

Western blotting was conducted as previously described [37]. The specific antibodies against β-catenin, STAT3, pSTAT3 (Y705), and GAPDH were purchased from Cell Signaling (Danvers, MA, USA).

### Gene knockdown

*STAT3* was knocked down using a short-hairpin RNA (shRNA)-expression lentivirus system containing the specific shRNA target sequences (1) GCAAAGAATCACATGCCACTT for HT29shSTAT3#1 and (2) GCACAATCTACGAAGAATCAA for HT29shSTAT3#2 in the vector pLKO.1-puro that was generated in 293 T cells. The procedure was the same as that in our previous study [37].

### Animal

Male NOD/SCID mice were purchased from BioLASCO Taiwan Co., Ltd., Taiwan. The 5-week-old mice were housed in a 12 h-light cycle at 22 °C. The animal studies were approved by the institutive ethical review committee in Mackay Memorial Hospital, Taiwan, which followed the NIH guidelines on the care and welfare of laboratory animals. Tumor xenografts were established by injecting 2 × 10^6^ HT29 (*n* = 3) or HT29shSTAT3#2 cells (*n* = 3), into the subcutaneous legs of mice of 5 weeks-old. The tumor sizes were measured using a digital caliper and recorded on day 14, 17, 19, 21 and 24. Tumor volume was recorded and calculated using the formula: 0.52 x width^2^ x length, herein the width represents the smaller tumor diameter.

### Statistical analysis

Statistical analyses were performed using GraphPad Prism V5.01 (GraphPad Software, Inc., CA, USA). All analytical data with more than two groups were evaluated using analysis of variance, followed by post hoc analysis with Bonferroni’s test. Student’s t-test was used to compare two groups. Additionally, *p* < 0.05 was considered an acceptable statistically significant difference.

## Results

### Formation of cancer stem-like tumorspheres from selective EGFR-positive CRCs

**Fig. 1:**
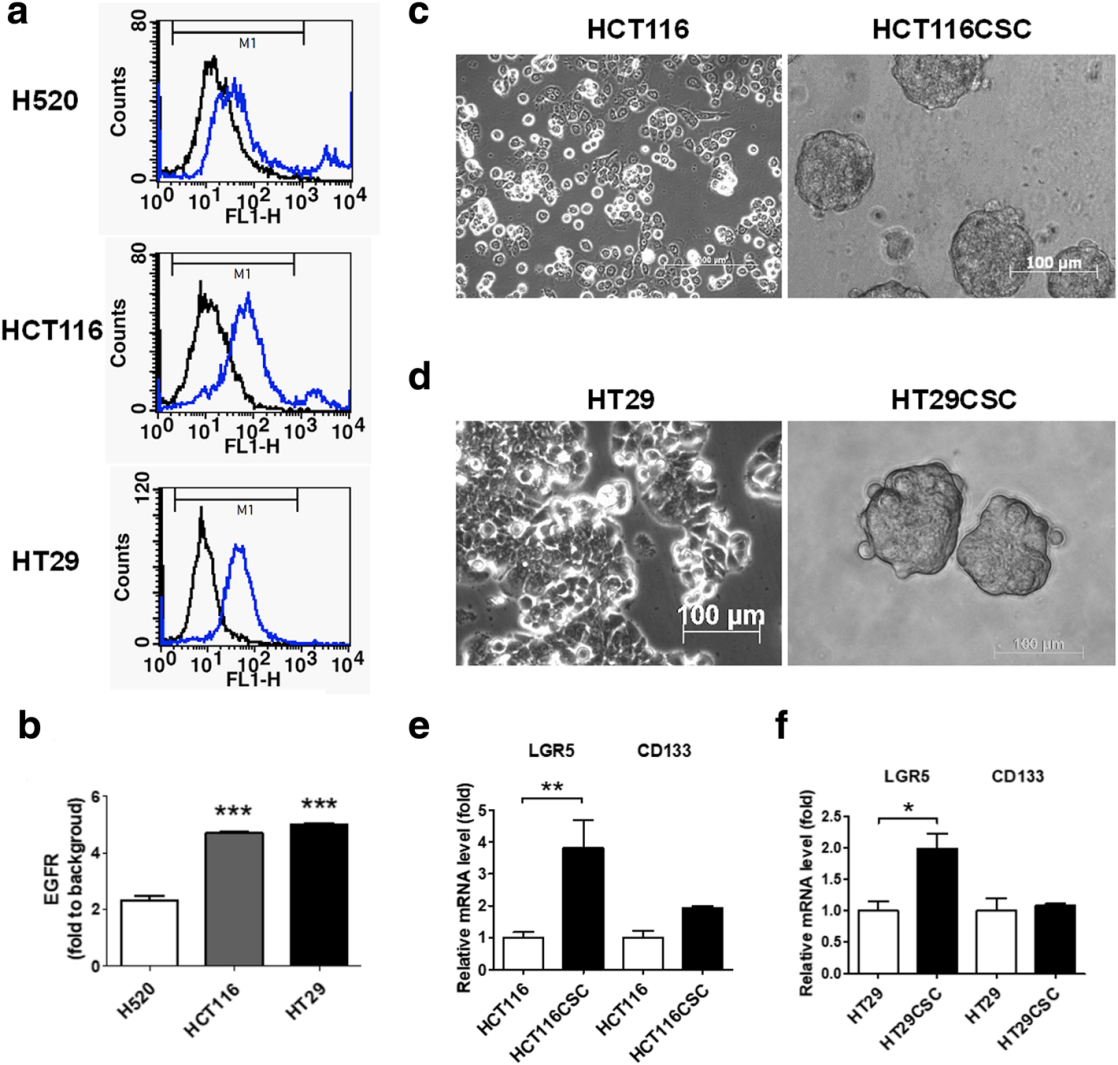
EGFR-positive CRC-derived tumorspheres mimicking CSCs as study models. (**a**) EGFR-positive CRC cells HCT116 and HT29 were selected. These cancer cells were validated for their EGFR expression through flow cytometry and compared with H520 cells, which are EGFR-negative. (**b**) Quantification of EGFR through fluoresce intensity revealed higher EGFR expression in HCT116 and HT29 cells compared with H520 cells. Therefore, HCT116 and HT29 were used as EGFR-positive models in this study. The tumorspheres derived from (**c**) HCT116 (HCT116CSC) and (**d**) HT29 (HT29CSC) were cultured in low-attachment six-well plates with serum-free medium containing 20 ng/mL of EGF, 20 ng/mL of fibroblast growth factor, 5 μg/mL of bovine insulin, and 4 μg/mL of heparin for 7 days to form tumorspheres measuring approximately 100 μm in diameter. (**e** and **f**) qPCR revealed higher *LGR5* expression in HCT116CSC and HT29CSC than in their respective parental cells; LGR5 is a known gastrointestinal stem cell marker. However, another stem cell marker CD133 was not significantly affected. Scale bar: 100 μm. *p < 0.05. **p < 0.01. ***p < 0.001

In this study, our first aim was to investigate the molecular mechanism of CSLCs derived from selective CRC cells that overexpressed EGFR, including HCT116 and HT29. The observed EGFR expression was validated through flow cytometry and compared with lung H520 cancer cells (Fig. 1a and b), which are EGFR-negative [37]. To analyze the CRC stem-like cells, four growth factors, namely EGF, bFGF, insulin, and heparin, were added to the HCT116 and HT29 cells and subsequently incubated. The culture methodology is described in the Materials and methods section. Both the HCT116 and HT29 cells formed tumorspheres within 7 days of incubation, reaching a size of > 100 μm (Fig. 1c and d). Both HCT116CSC and HT29CSC expressed higher *LGR5* levels without an increase in *CD133* (Fig. 1e and f). Because LGR5 is a marker of CSCs, the CSC-associated genes were proposed to be upregulated in the tumorspheres; therefore, these tumorspheres were used as the study models for investigating the molecular mechanism of CSCs.

### Activation of STAT3 in HCT116-derived tumorspheres

**Fig. 2:**
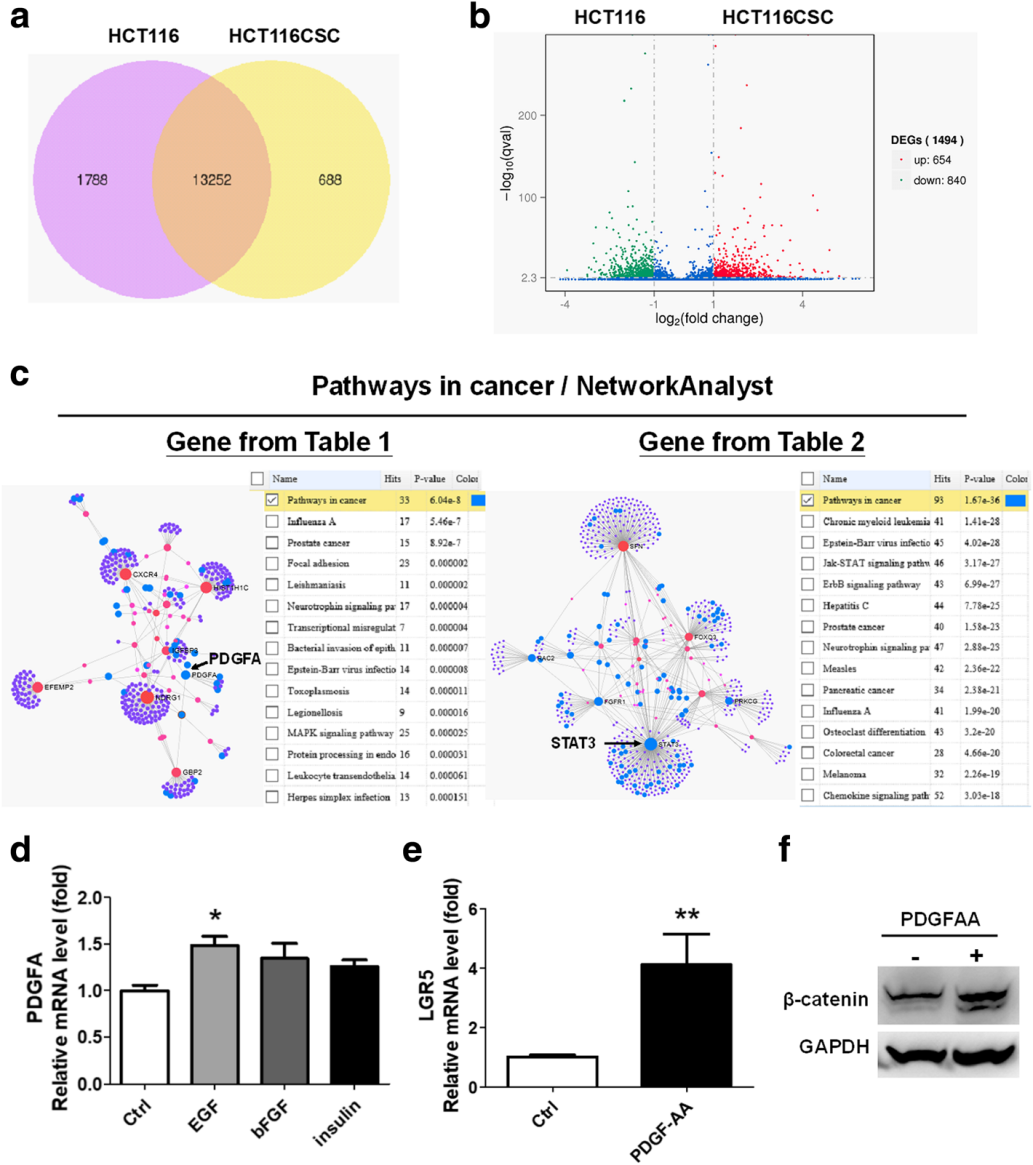
Gene expression profile of HCT116-derived tumorspheres investigated through RNAseq analysis indicated that PDGFA and STAT3 were significant. (**a**) In total, 688 genes increased and 1788 genes decreased in the HCT116-derived tumorspheres compared with the parental HCT116 cells. (**b**) Numbers of significant genes upregulated and downregulated in the HCT116-derived tumorspheres according to q value (*p* < 0.005 with > 1 fold change by log2) were 654 and 840, respectively. (**c**) Upregulated genes in the HCT116-derived tumorspheres were classified according to their molecular functions using PANTHER software (http://pantherdb.org/) and shown in Additional file 2: Figure S1. Upregulated genes with a > 3-fold change (log2) (Table 1) and genes associated with EGF, FGF, and insulin (Table 2) were analyzed using NetworkAnalyst (http://www.networkanalyst.ca/) for identifying the relevant signaling pathways based on the KEGG database. Genes associated with the cancer pathway are indicated by blue spots, revealing the overexpression of *PDGFA* and *STAT3*, indicated by arrows. (**d**) Through qPCR, the growth factor inducing *PDGFA* levels was identified. Thus, 20 ng/mL of EGF significantly induced *PDGFA* levels. (**e**) PDGF-AA was consequently demonstrated to be capable of inducing *LGR5* levels and (**f**) leading to β-catenin expression. *p < 0.05. **p < 0.01

We investigated the major driver genes in the formation and survival of CRC stem-like cells by conducting RNAseq analysis. The differential genes between the HCT116-derived tumorspheres and parental HCT116 cells were identified. In total, 688 genes were increased and 1788 genes were decreased in the HCT116-derived tumorspheres compared with parental HCT116 cells (Fig. 2a). According to the statistical q value (*p* < 0.005 with log2 fold change > 1), 654 and 840 genes were significantly upregulated and downregulated, respectively, in the HCT116-derived tumorspheres (Fig. 2b and Additional file 1: Table S1 present all the differential genes). Genes showing a > 3-fold change (log2) in expression are listed in Table 2. Genes showing a > 1-fold change (log2) in expression in the HCT116-derived tumorspheres were classified according to their molecular function by using the PANTHER classification system (Additional file 2: Figure S1). Among the significant genes, we were interested in the genes involved in the EGF, FGF, and insulin pathways because the tumorspheres were cultured using these growth factors. The EGF-, FGF-, and insulin-pathway-associated genes classified by PANTHER are shown in Table 3.

To analyze the interaction among the genes that showed a > 3-fold change (log2) in expression (Table 2) or genes associated with the EGF, FGF, and insulin pathways (Table 3), NetworkAnalyst was used. In NetworkAnalyst analysis, KEGG associated with cancer pathways showed that *PDGFA* was indicative (Fig. 2c) and *STAT3* may be involved in activating the formation of cancer stem-like tumorspheres (Fig. 2c). The inceased *PDGFA* levels were consequently validated in both HCT116CSC and HT29CSC through qPCR (Additional file 3: Figure S2). Furthermore, the HCT116 cells were treated with EGF, bFGF, and insulin individually to determine the growth factor playing a crucial role in inducing *PDGFA*; EGF was found to significantly induce the expressin of *PDGFA* in the HCT116 cells (Fig. 2d).

To demonstrate that *PDGFA* was significant in CRCs, we used the Kaplan–Meier plotter according to the PROGgeneV2 database. We found that higher levels of *PDGFA* were associated with poor survival in four cohorts of the clinical trial datasets GSE28814, GSE28722, GSE41258, and GSE29621 (Additional file 4: Figure S3) among the 12 available datasets. Moreover, 20 ng/mL of PDGF-AA added to the HCT116 cells was capable of inducing the expression of not only *LGR5* (Fig. 2e) but also β-catenin (Fig. 2f), a protein stabilized by Wnt, indicating that *PDGFA* overexpression in the cancer stem-like tumorspheres played a major role in inducing the activation of the Wnt signaling pathway. Furthermore, the Wnt-associated genes classfied using PANTHER are presented in Additional file 5: Table S2, revealing that Wnt7A, Wnt7B, Wnt9A, and others were upregulated in the tumorspheres, which indicates the activation of the Wnt signaling pathway.

### Therapeutic screening for identifying the potential agents against EGFR-positive CRCs

**Fig. 3:**
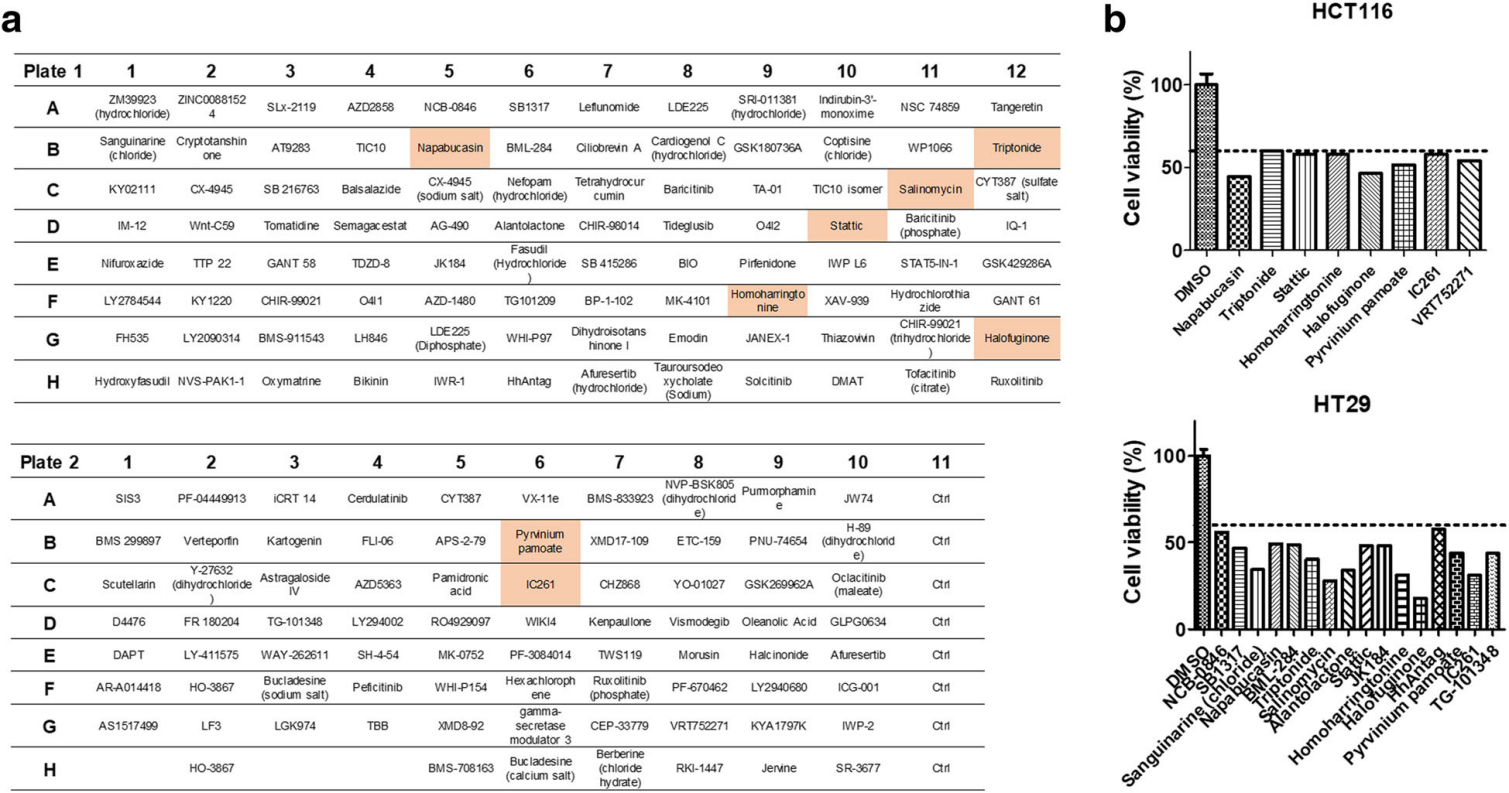
Therapeutic screening for identifying the specific agents against EGFR-positive HCT116 and HT29 cells. (**a**) To identify an efficient agent inhibiting EGFR-positive CRCs, a panel containing 172 inhibitory agents was used. Each inhibitor in the panel was added to HCT116 and HT29 cells individually followed by 48 h of incubation. The detailed cell viabilities are presented in Additional file 7: Table S3. In total, 8 of the 172 compounds reduced both HCT116 and HT29 cells by < 60% in vitro, which are marked in red*.* (**b**) Moreover, the inhibitors reducing the cell viability of HCT116 and HT29 cells by 60% individually are listed, revealing that the number of agents inhibiting HT29 cells was more than that of agents inhibiting HCT116 cells; HCT116 is a KRAS-mutant strain and HT29 is KRAS-normal

Because CSCs contribute to drug resistance and tumor recurrence, the Compound Screening Library (MedChemExpress, NJ, USA) targeting stem cells was used for identifying the potential therapeutics against EGFR-positive CRCs. A panel containing 172 compounds was added to HCT116 and HT29 cells separately at a dose of 1 μM and incubated for 48 h (Fig. 3a). The compounds that reduced the cell viabilities of both HCT116 and HT29 by 60% are marked in red. In total, 8 of the 172 compounds significantly reduced the viabilities of the two CRCs (Fig. 3a); the compounds targeted genes such as STAT3 (napabucasin, homoharringtonine, and stattic), Wnt (pyrvinium pamoate, triptonide, salinomycin, IC261), and transforming growth factor beta (TGFβ)/smad (halofuginone). The compound structures are shown in Additional file 6: Figure S4. The individual therapeutics against HCT116 and HT29 are illustrated in Fig. 3b, and the detailed experimental results are presented in Additional file 7: Table S3. The results indicated that the STAT3, Wnt, and TGFβ/smad signaling pathways were involved in the survival of the selective EGFR-positive CRCs.

### Targeting STAT3 inhibited the formation and survival of CRC stem-like tumorspheres

**Fig. 4:**
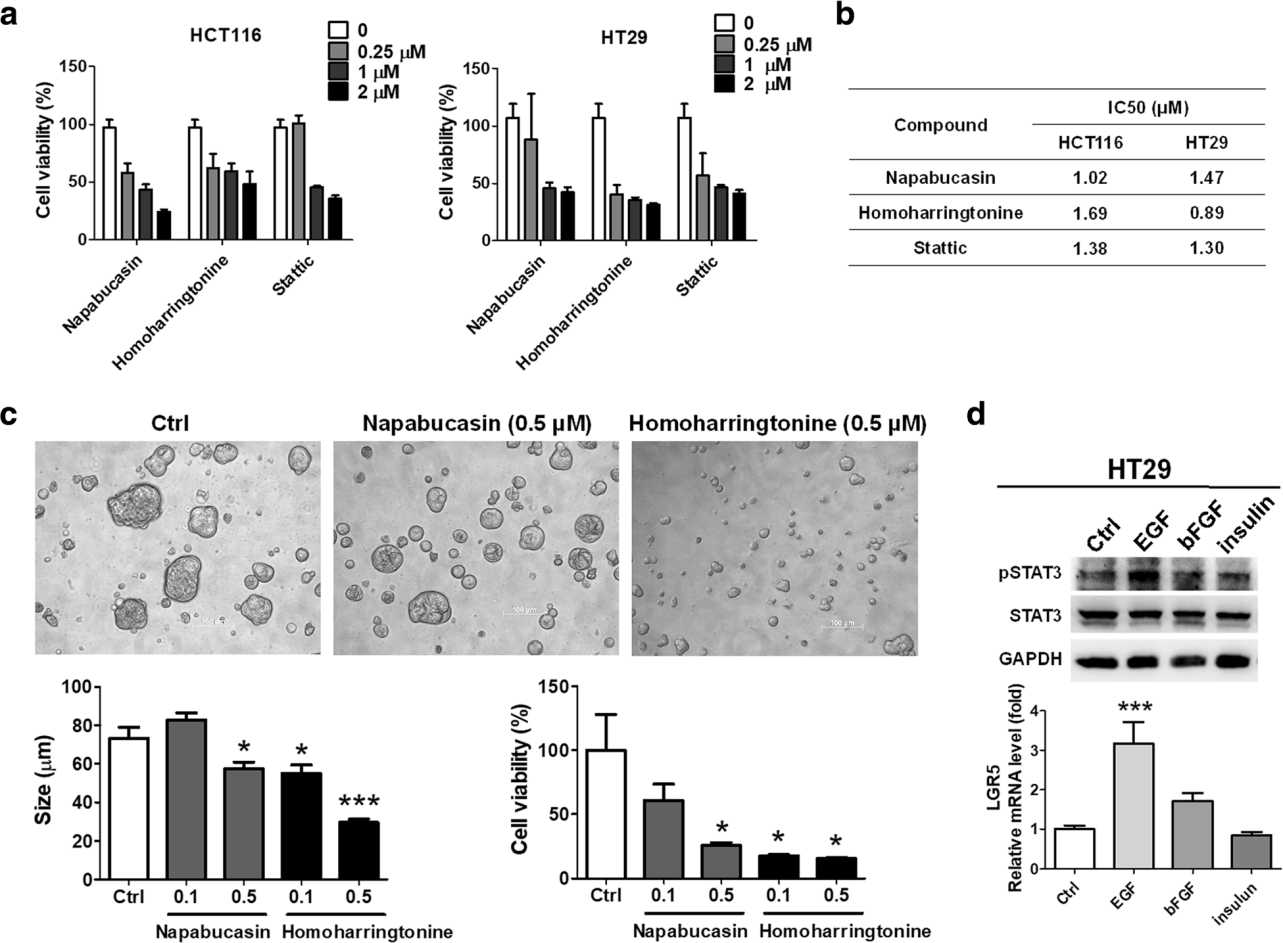
Targeting STAT3 with its specific inhibitors reduced the formation and survival of HT29-derived tumorspheres. (**a**) Inhibitors against STAT3 were selected because RNAseq analysis/NetworkAnalyst indicated STAT3 overexpression in the tumorspheres. First, the compounds were added to HCT116 and HT29 cells individually in a dose-dependent manner and incubated for 48 h. (**b**) The IC50 of each compound revealed that napabucasin inhibited HCT116 cells and homoharringtonine specifically inhibited HT29 cells. (**c**) Napabucasin and homoharringtonine were consequently investigated for their activities against the formation and survival of the HT29-derived tumorspheres by using 0.1 and 0.5 μM of compound and incubating for 7 days. The results indicated that napabucasin and homoharringtonine both significantly reduced the formation and survival of HT29CSCs; however, homoharringtonine priorly inhibited the HT29-derived tumorspheres. (**d**) To investigate the growth factors influencing STAT3 activation, Western blot analysis and qPCR were used for observing the STAT3 phosphorylation and stem cell marker *LGR5* levels. The results demonstrated that EGF played a major role in activating STAT3 and increasing *LGR5* levels. *p < 0.05. ***p < 0.001

To investigate and validate that STAT3 was involved in the formation and survival of CRC stem-like tumorspheres, compounds targeting STAT3 were used. Napabucasin, homoharringtonine, and stattic, which significantly reduced the cell viabilities of the HCT116 and HT29 cells, were measured for their inhibitory concentrations (IC50 values) against the HCT116 and HT29 cells in a dose-dependent manner from 0 to 2 μM (Fig. 4a). According to the IC50 results (Fig. 4b), napabucasin specifically inhibited HCT116 with an IC50 value of 1.02 μM and homoharringtonine specifically inhibited HT29 with an IC50 value of 0.89 μM. The results were consistent with the therapeutic screening results shown in Fig. 3b. Furthermore, napabucasin and homoharringtonine were added and investigated for their activities against the formation of HT29-derived tumorspheres. The results revealed that both napabucasin and homoharringtonine inhibited the formation of HT29-derived tumorspheres at a dose of 0.5 and 0.1 μM, respectively (Fig. 4c), including reducing the diameter (size, μm) and cell viability. We speculate that the lower inhibitory concentration against HT29-derived tumorspheres than against the parental HT29 cells was because the incubation time exceeded 7 days. Because the tumorspheres were cultured with the addition of the growth factors EGF, bFGF, and insulin, they were analyzed using Western blot analysis to identify the growth factor responsible for activating STAT3. EGF significantly induced STAT3 phosphorylation and elevated the mRNA levels of *LGR5* in the HT29 cells (Fig. 4d), implying that addition of EGF activated STAT3 in the cancer stem-like tumorspheres, thus contributing to the formation and survival of EGFR-positive CRC stem-like tumorspheres.

### STAT3 knockdown reduced expression of PDGFA and survival of HT29 cells and their derived tumorspheres through impairment of EGF-induced Wnt activation

**Fig. 5:**
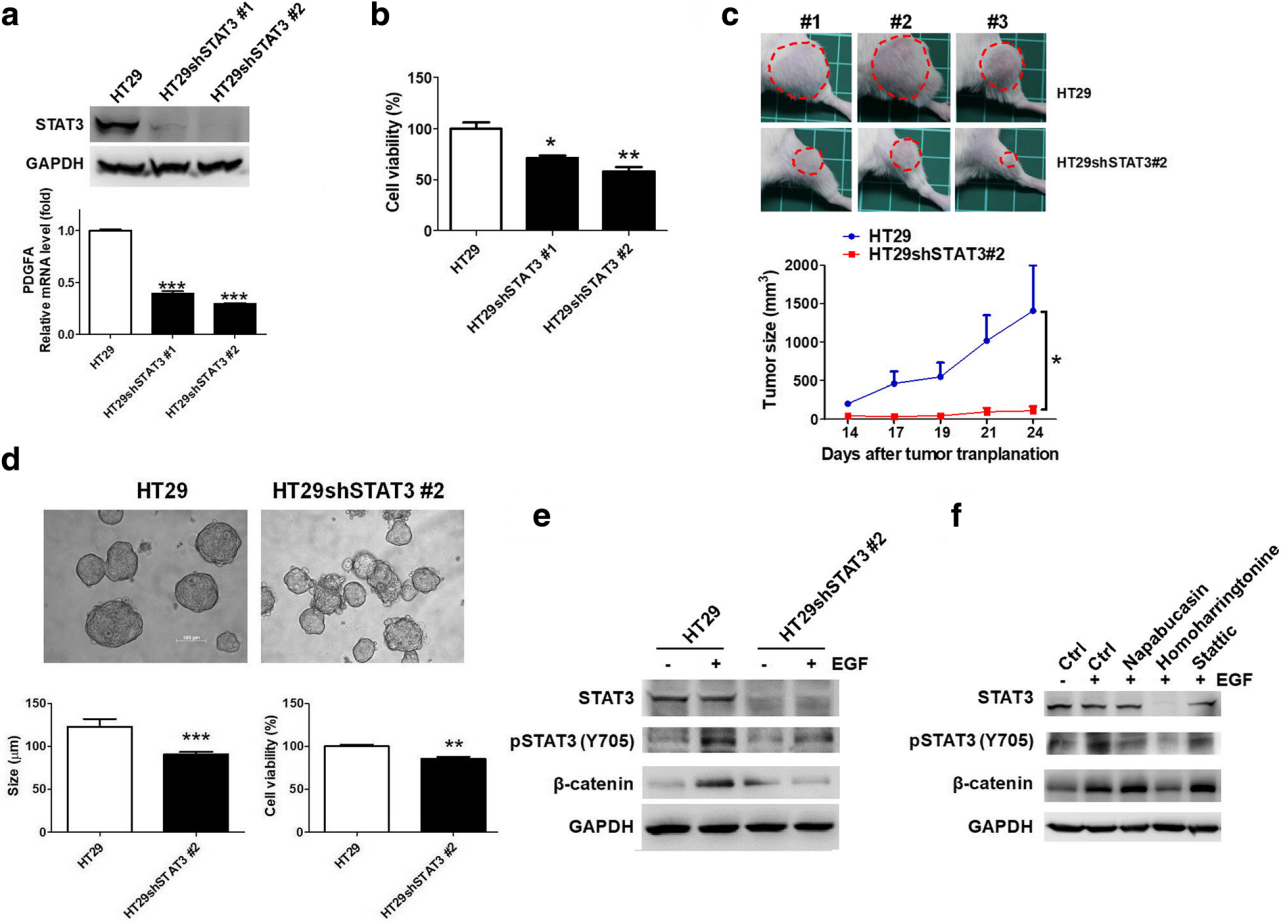
STAT3 knockdown reduced cell viability of HT29 cells and formation and survival of HT29-derived tumorspheres through inhibition of EGF-induced STAT3 phosphorylation. (**a**) STAT3 was knocked down using shRNA technique and measured using Western blot analysis, leading to the downregulation of *PGDFA* and (**b**) reduction of cell viability in HT29 cells. (**c**) We demonstrated that knockdown of STAT3 in HT29 cells (HT29shSTAT3#2) significantly reduced the tumor growth compared to parental HT29 cells in tumor xenografts (*n* = 3). Tumors are indicated by red circles. (**d**) To validate the involvement of STAT3 in the formation of cancer stem-like cells, the HT29 and HT29shSTAT3 cells cultured in serum-free medium with addition of EGF, bFGF, insulin, and heparin for 7 days were observed and investigated. The diameters of tumorspheres in the HT29shSTAT3 cells decreased compared with HT29 cells. In addition, the cell viability was reduced in the HT29shSTAT3 cells. (**e**) To verify whether STAT3 determines the activation of the Wnt signaling pathway, STAT3 phosphorylation and β-catenin expression were observed after treatment with 20 ng/mL of EGF for 2 h. The results demonstrated that EGF led to STAT3 phosphorylation and β-catenin overexpression in HT29 cells; however, there was no significant difference in HT29shSTAT3 cells, indicating that STAT3 determines the EGF-exacerbated Wnt signaling activation for the formation and survival of EGFR-positive cancer stem-like tumorspheres. (**f**) Consequently, to clarify the mechanism of homoharringtonine against the formation and survival of tumorspheres, STAT3 inhibitors were added to HT29 cells in 1 μM and incubated for 48 h. Homoharringtonine remarkably inhibited STAT3 expression and reduced EGF-mediated β-catenin expression. *p < 0.05. **p < 0.01. ***p < 0.001

To validate that STAT3 was involved in the formation and survival of the cancer stem-like tumorspheres, STAT3 was knocked down (Fig. 5a). However, STAT3 knockdown did not sufficiently inhibit the β-catenin expression (data not shown), a protein stabilized in Wnt signaling activation. An in-depth investigation revealed that the mRNA levels of *PDGFA* decreased in the HT29shSTAT3 cells (Fig. 5a). Furthermore, STAT3 knockdown significantly reduced the cell viability of the HT29 cells (Fig. 5b) and tumor growth in tumor xenografts (Fig. 5c). Thus, STAT3 determined cell survival in EGFR-positive colorectal tumors. Moreover, STAT3 knockdown inhibited the formation and survival of tumorspheres (Fig. 5d). To investigate whether Wnt signaling activation was regulated by the EGF–STAT3 pathway, 20 ng/mL of EGF was added to the HT29 and HT29shSTAT3 cells individually. The results indicated that EGF induced STAT3 phosphorylation, leading to β-catenin overexpression, which had no effect on the HT29shSTAT3 cells (Fig. 5e). Consequently, the HT29 cells were treated with the STAT3 inhibitors to clarify the mechanism of homoharringtonine, which significantly inhibited the formation and survival of tumorspheres (Fig. 4c). Only homoharringtonine remarkably inhibited STAT3 expression when the cells were treated with 1 μM of compound for 48 h (Fig. 5f), resulting in reduced EGF-mediated β-catenin expression. Thus, STAT3 is capable of exacerbating β-catenin and inducing PDGFA to promote Wnt signaling for maintaining the formation and survival of cancer stem-like tumorspheres in selective EGFR-positive CRCs.

## Discussion

This study conducted gene profiling analyses of colorectal HCT116CSCs through RNAseq analysis and determined that STAT3 played a crucial role in maintaining the CRC-stem-like tumorspheres. Furthermore, *PDGFA* overexpressed in the CSCs, specifically induced through the EGF–STAT3 pathway, increased the levels of *LGR5*, a marker of CRC stem cells, participating in the Wnt signaling pathway. Thus, EGF contributed to the survival of CSCs by STAT3 to activate the PDGFA-mediated Wnt signaling pathway in the selective EGFR-positive CRCs.

HCT116 has been demonstrated as an EGFR-overexpressed cell line [40, 41], possessing high stemness [42]. Because we previously demonstrated that EGFR may contribute to the formation of cancer stem-like tumorspheres in lung cancers [37], we assumed that EGFR may also contribute to cancer stemness in CRCs. To test the hypothesis, we selected EGFR-positive HCT116 and HT29 cell lines as study models for investigating the molecular mechanism of CSCs that were considered responsible for tumor metastasis and recurrence. We found that STAT3 activation (phosphorylation) and LGR5 mRNA levels were individually induced by EGF, revealing that EGFR is capable of exacerbating cancer stemness properties; this finding is consistent with that of a previous study [43]. We speculate that EGF induces EGFR phosphorylation [37], leading to signaling cascades and thus resulting in STAT3-mediated PDGFA overexpression and Wnt signaling activation. Because *LGR5* expression was induced by both EGF and PDGF-AA, we concluded that overexpressed PDGFA induced by EGF seems to be able to exacerbate CRC stemness.

Therapeutic screening using a panel containing 172 inhibitors against stem cells revealed that targeting the STAT3, Wnt, and TGFβ/smad pathways significantly reduced selective EGFR-positive CRC survival. Thus, inhibition of STAT3 expression using homoharringtonine and knockdown techniques validated that STAT3 was a driver gene contributing to the formation and survival of the CSLCs derived from the EGFR-positive CRCs. However, formation of cancer stem-like tumorspheres was still observed in the HT29shSTAT3 cells, although STAT3 expression was downregulated, as observed using Western blot analysis, indicating that blockade of STAT3 may be insufficient to completely eradicate CRCs. According to the therapeutic screening results, inhibitors targeting Wnt and TGFβ/smad should be of interest for future investigations of their activities against EGFR-positive CRCs. Anti-EGFR therapies, such as cetuximab and panitumumab, are suggested to cure patients with CRCs [44]. However, mutation of genes such as *KRAS, NRAS*, and *BRAF* in CRCs promotes cell proliferation and evokes drug resistance against anti-EGFR therapies [45]. Downstream signaling targets have been suggested for reducing drug resistance and preventing tumor recurrence. This study not only established the well-known STAT3 as a potential therapeutic target but also identified a specific anti-STAT3 agent, homoharringtonine, against EGFR-positive CRCs. Homoharringtonine, in particular, inhibited the formation and survival of the CSLCs through STAT3 expression downregulation, which resulted in a remarkable downregulation of EGF-mediated β-catenin. Homoharringtonine prevents the initial elongation step of protein synthesis [46]. However, Cao and collegues demonstrated that homoharringtonine also reduces pSTAT3 on Y705 in lung cancer cells [47]. We found that STAT3 knockdown reduced EGF-mediated β-catenin expression in this study, and this result is consistent with that of a previous study demonstrating that STAT3 mediated β-catenin in nine CRC cell lines [29]. Therefore, homoharringtonine probably reduced EGF-mediated β-catenin through primarily inhibiting STAT3 expression.

We also found that *PDGFA* overexpression was regulated by the EGF–STAT3 pathway, implying that the PDGF-AA binding receptor PDGFRαα served as another potential driver activating Wnt signaling, which could be a potential target against tumor recurrence [48]. A previous study indicated that PDGF-AA and PDGF-BB can express PDGFRs through an autocrine mechanism [49]. Therefore, although STAT3 was knocked down, causing downregulation of PDGFA and lower cell viability in the HT29 cells in this study (Fig. 5a, b, c), it was insufficient to inhibit β-catenin and completely block tumorsphere formation. Hence, we speculate that PDGFA is probably also induced by either bFGF or insulin (Fig. 2d) to lead to the autocrine mechanism of PDGF–PDGFR activation because PDGFR plays a crucial role in triggering and maintaining cancer stemness [50].

## Conclusion

Comparing whole gene expression profiles using PANTHER and NetworkAnalyst in combination with RNAseq, we determined the role of STAT3 activation in CRC-derived cancer stem-like tumorspheres. Additionally, we validated that *PDGFA* increased in the tumorspheres through the EGF–STAT3 pathway, leading to the induction of *LGR5* and Wnt signaling. The therapeutic screening and knockdown experiment confirmed the observation, indicating that STAT3 targeting is a potential treatment strategy against EGFR-positive CRCs. This study not only elucidated the molecular mechanism of CSLCs but also provided responsible targeted inhibitors such as homoharringtonine against selective EGFR-positive CRCs.

## Additional files

Additional file 1: **Table S1.** All the differential genes analyzed using RNAseq shown in the attached excel file. (XLS 902 kb)

Additional file 2: **Figure S1.** Differentially upregulated genes were classified using PANTHER, showing the gene number in distinguished functions. (TIF 161 kb)

Additional file 3: **Figure S2.** *PDGFA* levels were validated to be upregulated in the tumorspheres compared with their parental cells derived from EGFR-positive HCT116 and HT29 cells. (TIF 47 kb)

Additional file 4: **Figure S3.** Higher levels of *PDGFA* are associated with overall survival in patients with CRC in the database PROGgeneV2. (TIF 226 kb)

Additional file 5: **Table S2.** Upregulated genes associated with the Wnt signaling pathway in HCT116-derived tumorspheres (analyzed using PANTHER, http://pantherdb.org/). (DOCX 14 kb)

Additional file 6: **Figure S4.** Chemical structures of the eight compounds that significantly reduced the cell viabilities of both HCT116 and HT29 cells by 60%. (TIF 287 kb)

Additional file 7: **Table S3.** Detailed measurement of cell viability in therapeutic screening against HCT116 and HT29 cells. (DOCX 632 kb)
