# Supplementary figures and images for: STAT3 exacerbates survival of cancer stem-like tumorspheres in EGFR-positive colorectal cancers: RNAseq analysis and therapeutic screening
Source: J Biomed Sci. 2018 Aug 2;25:60. doi: 10.1186/s12929-018-0456-y (PMC6090986; doi:10.1186/s12929-018-0456-y)

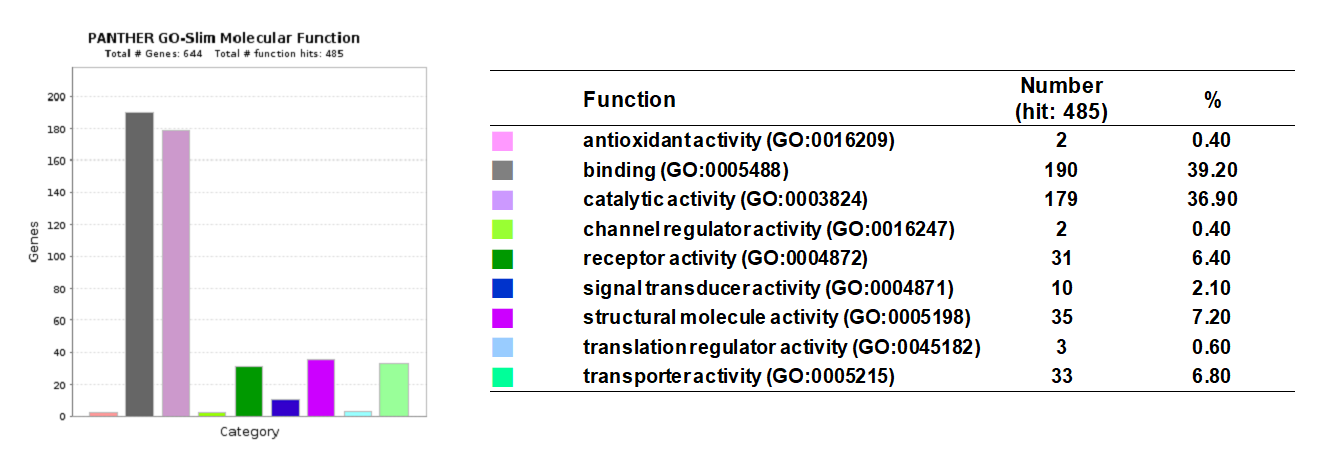

Supplement: Supplementary file 2 — Figure S1. Differentially upregulated genes were classified using PANTHER, showing the gene number in distinguished functions. (TIF 161 kb) [file 12929_2018_456_MOESM2_ESM.tif]

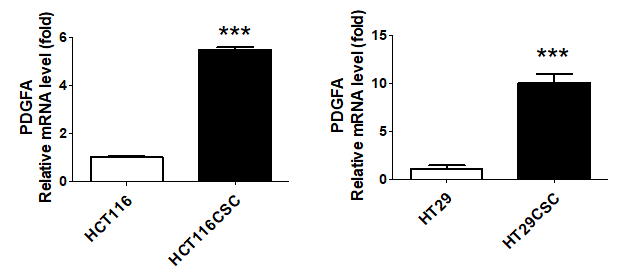

Supplement: Supplementary file 3 — Figure S2. PDGFA levels were validated to be upregulated in the tumorspheres compared with their parental cells derived from EGFR-positive HCT116 and HT29 cells. (TIF 47 kb) [file 12929_2018_456_MOESM3_ESM.tif]

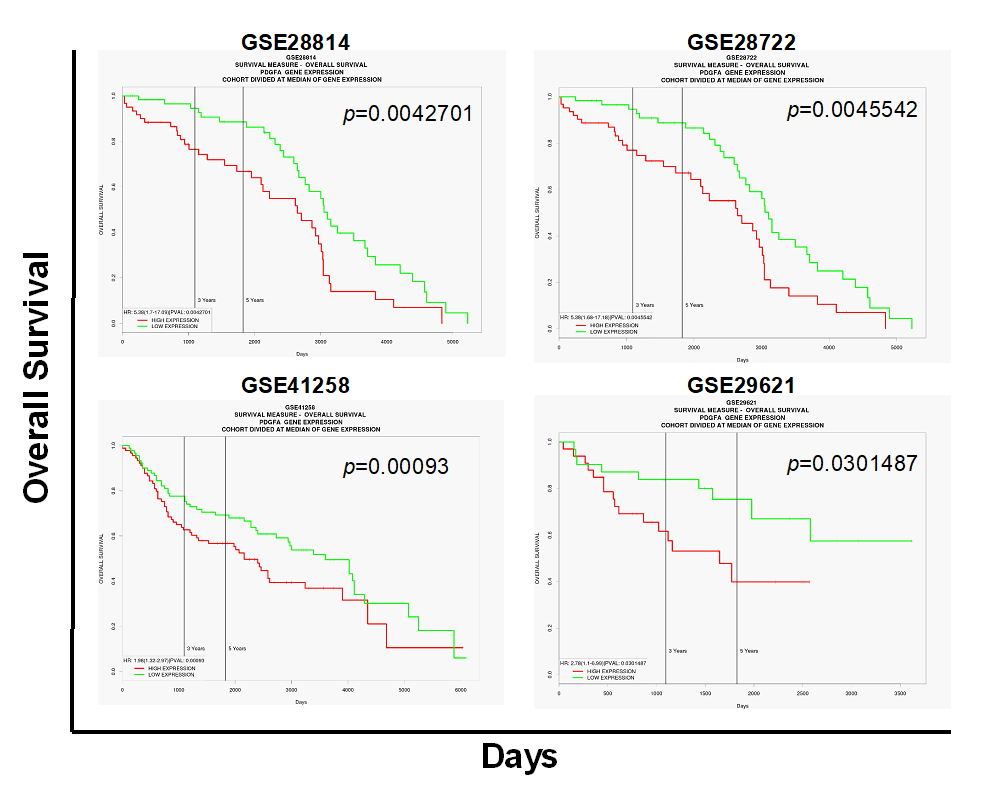

Supplement: Supplementary file 4 — Figure S3. Higher levels of PDGFA are associated with overall survival in patients with CRC in the database PROGgeneV2. (TIF 226 kb) [file 12929_2018_456_MOESM4_ESM.tif]

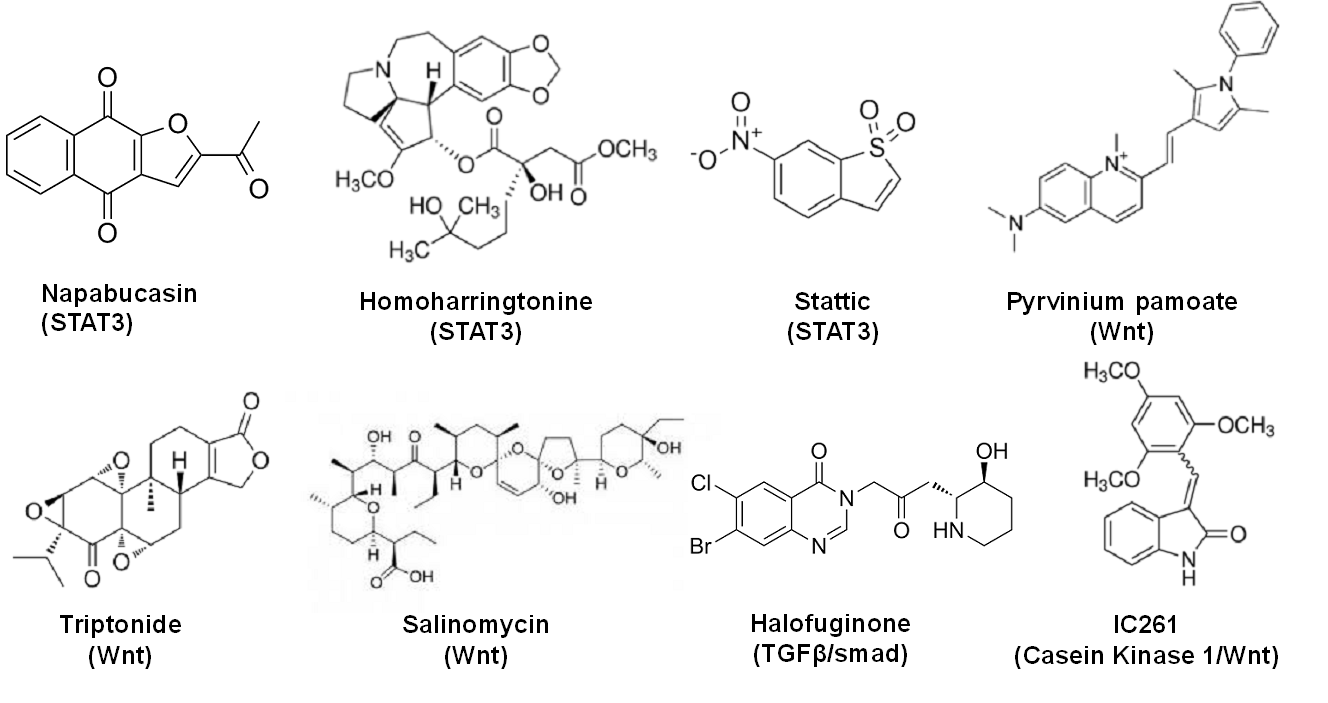

Supplement: Supplementary file 6 — Figure S4. Chemical structures of the eight compounds that significantly reduced the cell viabilities of both HCT116 and HT29 cells by 60%. (TIF 287 kb) [file 12929_2018_456_MOESM6_ESM.tif]
