# Supplementary material for: STAT3 exacerbates survival of cancer stem-like tumorspheres in EGFR-positive colorectal cancers: RNAseq analysis and therapeutic screening
Source: J Biomed Sci. 2018 Aug 2;25:60. doi: 10.1186/s12929-018-0456-y (PMC6090986; doi:10.1186/s12929-018-0456-y)
Supplement: Supplementary file 5 — Table S2. Upregulated genes associated with the Wnt signaling pathway in HCT116-derived tumorspheres (analyzed using PANTHER, http://pantherdb.org/). (DOCX 14 kb) [file 12929_2018_456_MOESM5_ESM.docx]

**Table S2. Upregulated genes associated with the Wnt signaling pathway in HCT116-derived tumorspheres (analyzed using PANTHER, http://pantherdb.org/)**

| **Pathway** | **Gene** | **fold chang (log2)** | **qvalue** |
| --- | --- | --- | --- |
| Wnt | PLCB4 | 2.1345 | 0.00368 |
|  | WNT7B | 1.8121 | 1.54E-11 |
|  | GNG7 | 1.1313 | 0.002593 |
|  | WNT9A | 1.5631 | 5.88E-06 |
|  | WNT7A | 2.8391 | 0.000195 |
|  | PPARD | 1.2704 | 1.75E-10 |
|  | PPP2R5B | 2.4064 | 1.95E-14 |
|  | PRKCG | 2.2982 | 0.004287 |
|  | CELSR3 | 1.2745 | 0.0013 |
|  | TLE2 | 1.1837 | 2.36E-06 |
