# Supplementary material for: STAT3 exacerbates survival of cancer stem-like tumorspheres in EGFR-positive colorectal cancers: RNAseq analysis and therapeutic screening
Source: J Biomed Sci. 2018 Aug 2;25:60. doi: 10.1186/s12929-018-0456-y (PMC6090986; doi:10.1186/s12929-018-0456-y)

**Table S3. Detailed measurement of cell viability in therapeutic screening against HCT116 and HT29 cells**


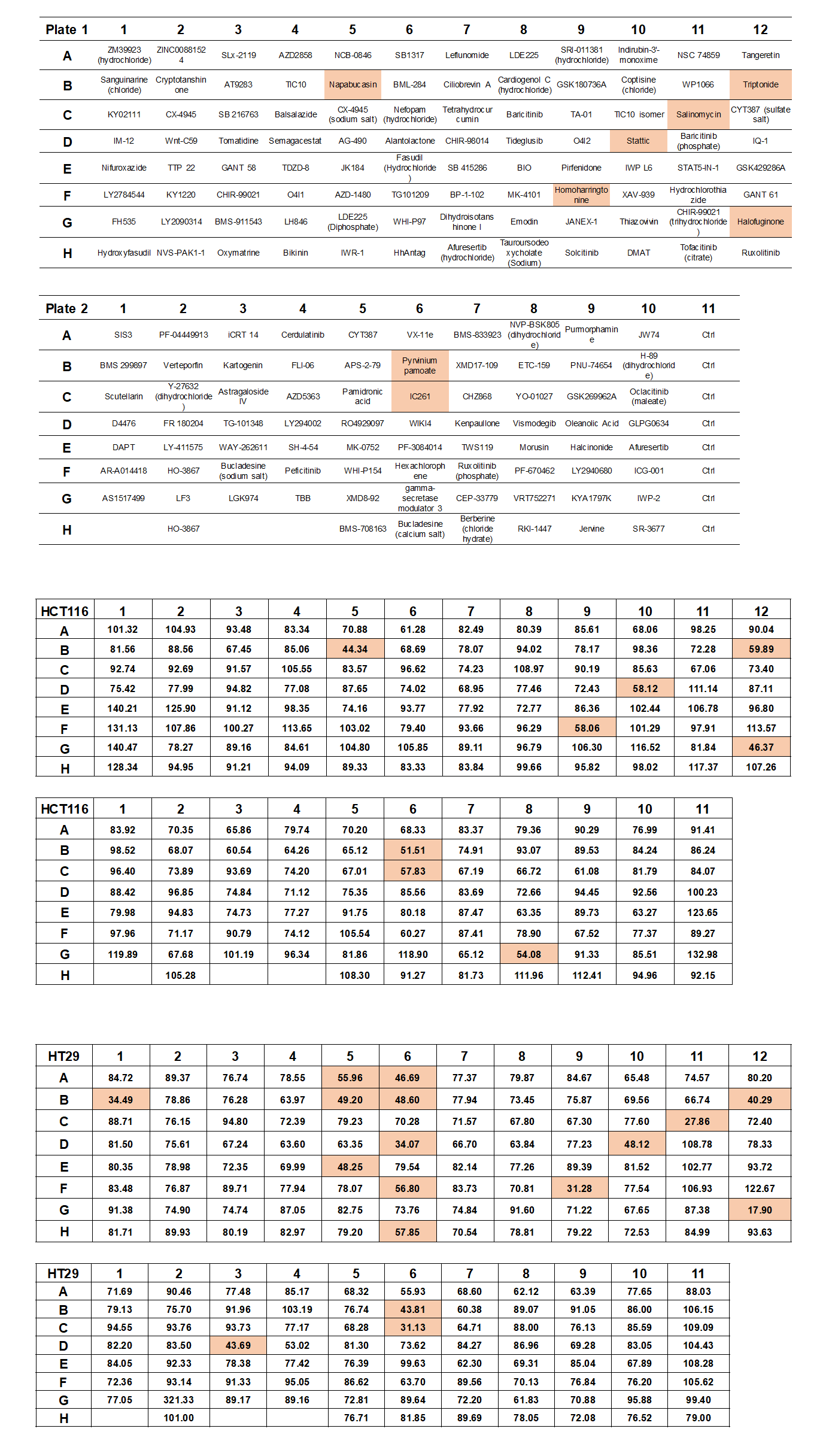

Supplement: Supplementary file 7 — Table S3. Detailed measurement of cell viability in therapeutic screening against HCT116 and HT29 cells. (DOCX 632 kb) [file 12929_2018_456_MOESM7_ESM.docx]
